# Overcoming Biomass Recalcitrance by Combining Genetically Modified Switchgrass and Cellulose Solvent-Based Lignocellulose Pretreatment

**DOI:** 10.1371/journal.pone.0073523

**Published:** 2013-09-27

**Authors:** Noppadon Sathitsuksanoh, Bin Xu, Bingyu Zhao, Y.-H. Percival Zhang

**Affiliations:** 1 Biological Systems Engineering Department, Virginia Tech, Blacksburg, Virginia, United States of America; 2 Horticulture Department, Virginia Tech, Blacksburg, Virginia, United States of America; 3 Institute for Critical Technology and Applied Sciences (ICTAS), Virginia Tech, Blacksburg, Virginia, United States of America; 4 DOE BioEnergy Science Center (BESC), Oak Ridge, Tennessee, United States of America; 5 Gate Fuels Inc., Blacksburg, Virginia, United States of America; 6 Cell-Free Bioinnovations Inc, Blacksburg, Virginia, United States of America; Nanjing Agricultural University, China

## Abstract

Decreasing lignin content of plant biomass by genetic engineering is believed to mitigate biomass recalcitrance and improve saccharification efficiency of plant biomass. In this study, we compared two different pretreatment methods (i.e., dilute acid and cellulose solvent) on transgenic plant biomass samples having different lignin contents and investigated biomass saccharification efficiency. Without pretreatment, no correlation was observed between lignin contents of plant biomass and saccharification efficiency. After dilute acid pretreatment, a strong negative correlation between lignin content of plant samples and overall glucose release was observed, wherein the highest overall enzymatic glucan digestibility was 70% for the low-lignin sample. After cellulose solvent- and organic solvent-based lignocellulose fractionation pretreatment, there was no strong correlation between lignin contents and high saccharification efficiencies obtained (i.e., 80–90%). These results suggest that the importance of decreasing lignin content in plant biomass to saccharification was largely dependent on pretreatment choice and conditions.

## Introduction

Biomass recalcitrance to saccharification is one of the major obstacles to cost-efficient production of biofuels and value-added biochemicals from lignocellulose. Biomass recalcitrance is attributed to many factors, such as low substrate accessibility, high degree of polymerization of cellulose, the presence of lignin and hemicellulose, high crystallinity, large particle size, poor porosity, and so on [1], [2]. Several of these factors (e.g., particle size, porosity, crystallinity, and lignin) are closely related to cellulose accessibility to cellulase, which has been recognized as the most important substrate factor that limits efficient enzymatic hydrolysis of pretreated biomass [3]–[5].

Lignin is believed to block cellulose accessibility to cellulase and decreases cellulase activity by competitively binding to hydrolytic enzymes [6]. Consequently, a large quantity of costly enzymes is required to achieve acceptable enzymatic saccharification efficiencies. Therefore, tremendous research efforts have been focused on decreasing lignin content in bioenergy crops [7], [8]. For example, down-regulation of lignin biosynthesis enzymes in alfalfa decreased plant lignin content, resulting in improved enzymatic saccharification efficiency with dilute sulfuric acid pretreated biomass [9]. Furthermore, low-lignin transgenic plant samples may require less severe pretreatment conditions (i.e., lower energy consumption).

Switchgrass (*Panicum virgatum* L.) has been regarded as a promising bioenergy crop in North America. Transgenic switchgrass plants with lower lignin contents have been achieved [10]–[12]. These reduced lignin transgenic plants were reported to have uncompromised biomass yield compared to wild type plants under controlled growth conditions [10], [11].

Dilute acid (DA) pretreatment, the most widely investigated pretreatment, effectively depolymerizes and solubilizes the most labile biomass components i.e. hemicelluloses, providing cellulose-lignin-rich solids, which can be hydrolyzed by cellulase [13]–[15]. A high enzyme loading, however, is usually required to achieve high soluble sugar yields mainly due to non-specific binding of cellulase to lignin [14]. Cellulose solvent and organic acid-based lignocellulose fractionation (COSLIF) has been recognized as an efficient means to disrupt highly ordered hydrogen bonding in cellulose chains in biomass [16]. The resulting solids are extremely reactive and highly accessible to cellulase, leading to very high sugar yields, even at low cellulase loadings [4], [14].

To further investigate the combinatorial effect of lignin content and pretreatment method on downstream scarification efficiencies, we compared the saccharification efficiencies of DA pretreatment and COSLIF on multiple transgenic plant lines, whose lignin contents were regulated by controlling the expression of a lignin synthesis gene *4-coumarate: COA ligase* (*4cl*) [11].

## Materials and Methods

### Chemicals and materials

All chemicals were reagent grade and purchased from Sigma-Aldrich (St. Louis, MO), unless otherwise noted. Phosphoric acid (85% w/w), sulfuric acid (95% w/w), ethanol (95% v/v) were purchased from Fisher Scientific (Houston, TX). The *Trichoderma reesei* cellulase (Novozyme® 50013) and β-glucosidase (Novozyme® 50010) were gifted by Novozymes North America (Franklinton, NC). They had activities of 84 filter paper units (FPU) of cellulase per mL and 270 units of β-glucosidase per mL. The naturally dried switchgrass samples were milled into small particles by a Pallmann counter-rotating knife ring flaker (Clifton, NJ). The resulting particulates with nominal sizes of 40–60 mesh (250–400 µm) were used for pretreatment experiments.

### Switchgrass feedstock biomass

RNAi:*Pv4CL1* low lignin transgenic switchgrass was generated by *Agrobacterium*-mediated genetic transformation [11]. All transgenic plants and wild-type plants used in this study were originally derived from the same batch seeds which are from selected cv. Alamo lines HR8 (♀) × HR7 (♂). Therefore, their genetic backgrounds were highly similar to each other. All plants used in this study were grown under the same condition in the horticulture greenhouse at Virginia Tech, with the temperature set to 22°C at night and 28°C during the day with a 12–14 hour light. The plants were grown in Miracle-Gro Potting Mix (Miracle-Gro Lawn Products, Inc., Marysville, OH) in 1.1×10^−2^ m^3^ pots and watered twice a week. To further eliminate the tissue culture effects on the growth of transgenic plants, the plants were vegetatively propagated for 2 years. All plant samples were harvested when 50% of the tillers had flowered in the 2^nd^ year. Three wild type plants were harvested and pooled together to minimize variations.

### Fluorescence microscopy and histological staining

The internodes of the WT and T_1_ plants were embedded in 2.5% agarose and cut with a Leica VT1200 vibrating blade microtome (Leica Microsystems Inc., Buffalo Groove, IL) into 50 µm thick sections. Phloroglucinol staining of the 50 µm thick stem sections were used to analyze the lignin deposition patterns by visualization under an Olympus SZXZ-RFL3 fluorescence microscope (Olympus America, Melville, NY) [17].

### COSLIF procedure

The COSLIF-pretreated switchgrass was prepared as described previously [14]. One gram of switchgrass was mixed with 8 mL of 85% (w/w) H_3_PO_4_ at 50°C and atmospheric pressure for 45 min. The switchgrass/phosphoric acid slurry was mixed with 20 mL of 95% (v/v) ethanol to stop the reaction. Solid-liquid separation was conducted in a swing bucket centrifuge at 4500 rpm at room temperature for 10 min. After the supernatant was discarded, the pellets were suspended in 40 mL of 95% (v/v) ethanol. After centrifugation, the solid pellets were washed by 80 mL of deionized water. After centrifugation, the remaining solid pellet was neutralized to pH ∼6 with 2 M sodium carbonate.

### DA pretreatment

The DA pretreatment was conducted using 1.3% (w/w) H_2_SO_4_ at a solid loading of 10% (w/w) at 130°C and ∼20 psi (autoclave, Consolidated Sterilizer Systems, Boston, MA) for 40 min [9]. After DA, the hydrolysate was separated by centrifugation. The switchgrass residue was washed in water prior to enzymatic hydrolysis. All experiments were conducted in triplicate.

### Carbohydrate and lignin assays

The structural carbohydrate composition of lignocellulose was determined by a modified quantitative saccharification procedure [18]. Monomeric sugars were measured by a Shimadzu HPLC with a Bio-Rad Aminex HPX-87H column (Richmond, CA) equipped with refractive index detector. The concentrations of glucose and xylose were measured in enzymatic hydrolysate, whereby galactose and mannose were co-eluted with xylose. The column was operated with 5 mM H_2_SO_4_ as a mobile phase at 60°C and a flow rate of 0.6 mL/min [4], [14].All experiments were conducted in triplicate. Lignin contents of switchgrass samples were determined according to standard NREL protocol [19], where both acid insoluble and acid soluble lignin values were obtained. The acid soluble lignin content of switchgrass samples was determined by using UV absorbance at 240 nm and absorptivity of 25 L/g cm.

### Determination of lignin aromatic units by thioacidolysis

Whole stems of different plants were dried and treated for thioacidolysis followed by gas chromatography (GC)-mass spectrometer (MS) to measure the monolignol composition. Extractive-free lignin was obtained by acetone extraction in a Soxhlet apparatus for 24 h. The dried lignin of each sample was silylated prior to thioacidolysis [20]. In short, the silylated sample was injected into the GC column (Restek RTX5-MS, 1 µm film thickness, 30 m×3.2 mm i.d., Thames Restek UK Ltd., Windsor, UK). The GC-MS analysis was modified from a previous method [21] and performed on a VG 70SE double-focusing magnetic sector instrument, interfaced to a Hewlett-Packard (HP) 5790 GC (Hewlett-Packard Co., Fullerton, CA).

### Enzymatic hydrolysis

The COSLIF- and DA-pretreated samples were diluted to 20 g glucan per liter in a 50 mM sodium citrate buffer (pH 4.8) supplemented with 0.1% (w/v) NaN_3_. The enzyme loadings were 5 filter paper units (FPU) per gram of biomass and 10 units of β-glucosidase per gram of biomass, otherwise noted. Hydrolysis experiments were carried out in a rotary shaker at 250 rpm at 50°C for 24 h and 72 h for COSLIF- and DA-pretreated switchgrass samples, respectively. Hydrolysis reaction mixtures were subjected to centrifugation to separate supernatant from unhydrolyzed solids. Supernatants were left at room temperature for 30 min to allow a complete conversion of cellobiose to glucose. Then supernatants were acidified with 1% (w/w) H_2_SO_4_ prior to HPLC analyses for monomeric sugar releases.

## Results and Discussion

**Figure 1:**
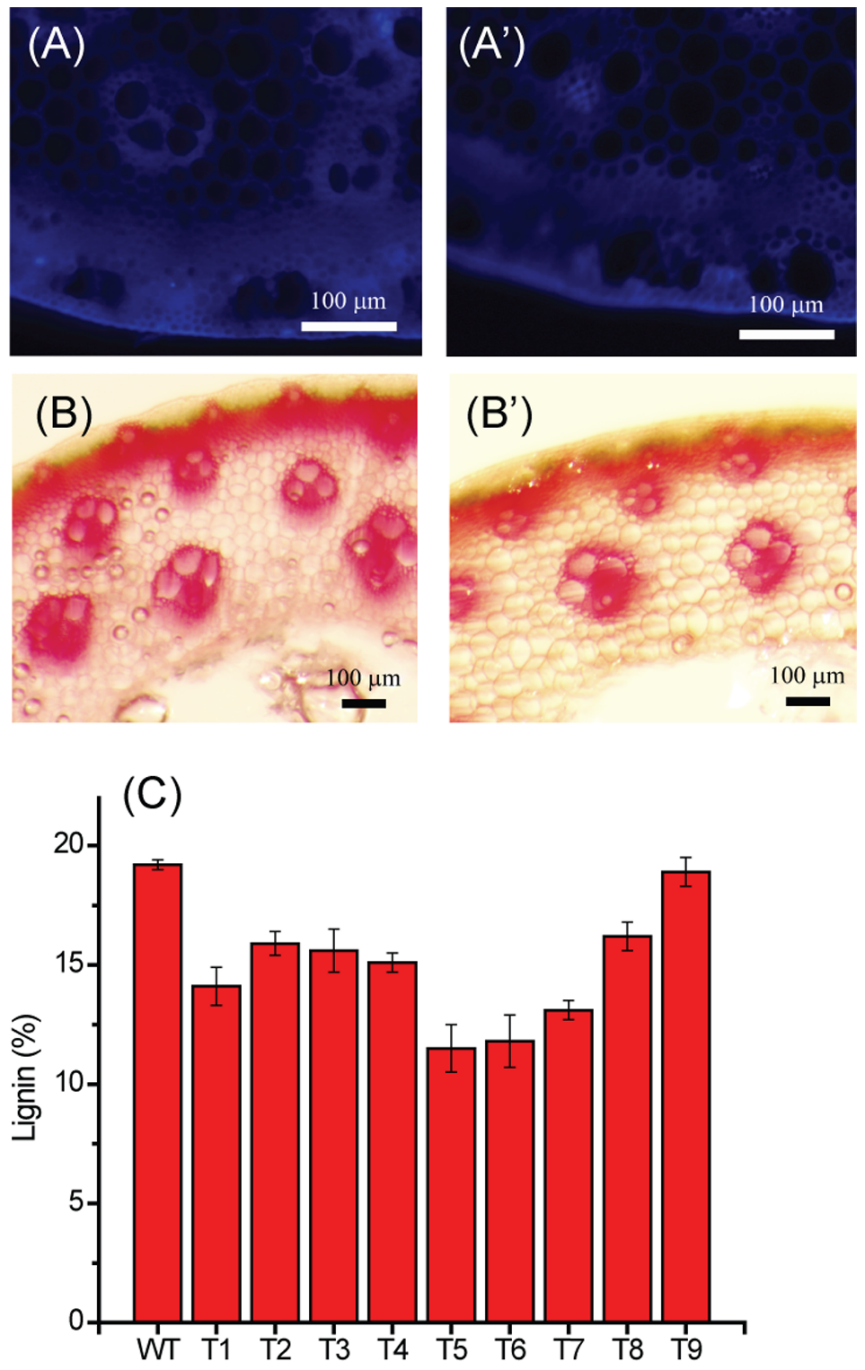
Lignin autofluorescence (A and A'), histological staining by phloroglucinol-HCl reagents (B and B'). Analysis of wild-type and transgenic switchgrass lines. Lignin content of wild type and transgenic switchgrass lines (C).

Impact of *Pv4CL1* suppression on lignin content of transgenic plants was initially screened by autofluorescence and phloroglucinol staining of transverse stem sections [11], [17]. The screening result showed that transgenic lines had varied degrees of impaired lignifications (**Fig. 1A–B**). The lignin contents of nine selected T_1_ transgenic plants and pooled wild type samples were further measured according to the standard NREL protocol. The result showed that transgenic plants had lignin contents ranging from 12 to 19%, lower than the wild type plant (19.2%) (**Fig. 1C**). Such genetic modification did not impair biomass productivity (**Table 1**). The composition of lignin aromatic units of transgenic plants was also changed with decreased G:S ratios **(** **Table 1**). A decrease in lignin content in transgenic plants was inversely proportional to carbohydrate levels of the plant biomass, as shown in an increase in carbohydrate/lignin ratio **(** **Table 1**).

**Table 1:** Growth performance, carbohydrate content, and lignin aromatic units of transgenic (T_0_) and wild-type (WT) plants.

Switchgrass T_0_ Lines	Total dried weight (g)	Carbohydrates (wt. %)		Lignin (wt. %)			Carbohydrate /lignin ratio	Lignin aromatic units (%)			G:S ratio
		Glucan	Xylan	Acid- insoluble	Acid- soluble	Total lignin		H	G	S	
Wild type	251.0±27.3	34.8±0.4	17.6±0.3	18.5±0.0	0.7±0.2	19.2±0.2	2.72	0.23±0.09	11.60±0.18	7.37±0.27	1.57
T_0_ −1	224.9±9.4	35.4±1.2	14.6±0.9	14.2±0.4	1.7±0.3	15.9±0.5	3.14	0.30±0.23	8.58±1.04	7.01±0.95	1.22
T_0_ −2	227.7±47.6	36.9±0.5	19.3±0.0	14.2±0.9	1.4±0.2	15.6±0.9	3.60	1.48±0.23	7.20±0.69	6.92±0.92	1.04
T_0_ −3	226.0±20.9	34.7±0.8	18.8±0.6	13.8±0.2	1.3±0.4	15.1±0.4	3.54	1.41±0.35	7.08±0.08	6.61±0.39	1.07
T_0_ −4	258.2±11.3	39.2±0.1	23.0±0.3	12.2±0.2	0.9±0.3	13.1±0.4	4.75	1.11±0.19	3.60±0.22	8.39±0.03	0.43

**Figure 2:**
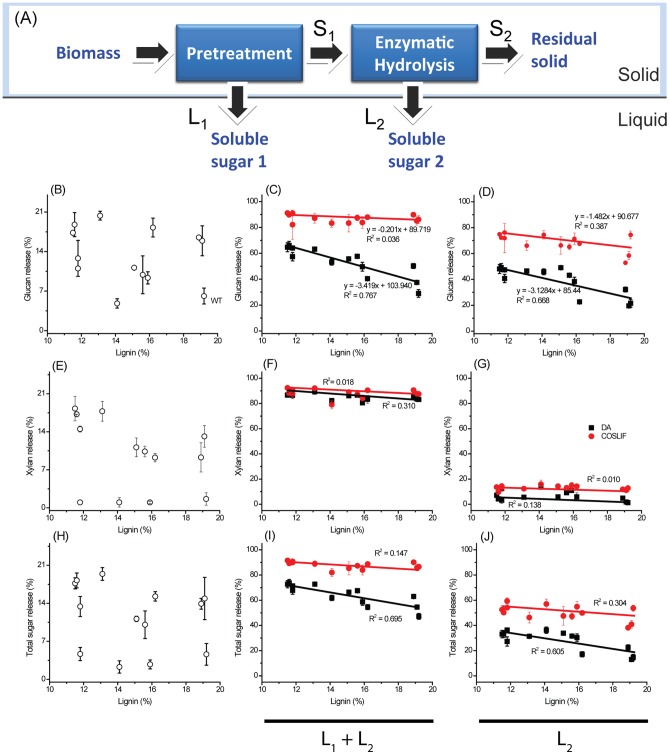
Schematic diagram of mass balance (A), the relationship between lignin content and glucan releases before and after pretreatments (B, C, and D), xylan releases before and after pretreatments (E, F, and G), and overall sugar release before and after pretreatments (H, I, and J). The correlation between lignin contents and glucan, xylan, and total sugar releases by pretreatment and enzymatic hydrolysis (L_1_+L_2_) is shown in the middle column. The correlation between lignin contents and glucan, xylan, and total sugar releases by enzymatic hydrolysis (L_2_) is shown in the left column. Enzymatic hydrolysis was carried out at 20 g biomass/L. All pretreated solids were hydrolyzed at 5 FPU of cellulase and 10 units of β-glucosidase per gram of biomass. DA condition was 130°C and 20 psi for 40 min. COSLIF condition was 50°C and atmospheric pressure for 45 min. DA- and COSLIF-pretreated biomass samples were hydrolyzed by cellulase for 72 h and 24 h, respectively. Data are from triplicate.

WT and transgenic plants, without pretreatment, were hydrolyzed by commercial cellulase supplemented with β-glucosidase. The enzymatic glucan releases of most transgenic plants were higher than that of WT, indicating less recalcitrance in transgenic plants. However, there was no correlation between lignin level and enzymatic glucan release (**Fig. 2B**).

Pretreatment was applied to investigate the combinatory effect of genetically modified switchgrass and pretreatments. All biomass samples were pretreated with DA and COSLIF. Sugar releases from pretreatment (stream L_1_) and enzymatic hydrolysis (stream L_2_) were determined from all samples (**Fig. 2A**). The overall glucan release (stream L_1_+ stream L_2_) and enzymatic glucan release (stream L_2_) were negatively correlated with lignin levels in DA-pretreated samples (**Fig. 2C and 2D**). The highest overall glucan release and enzymatic glucan release values were 70% and 50%, respectively for the biomass sample containing 11.0 wt.% lignin compared to 6% enzymatic glucan release of WT (19.2 wt.% lignin).

Shi et al [15] investigated DA pretreatment at 140, 160, and 180°C for 1–60 min and found that partition of sugar release in stream L_1_ and stream L_2_ depends on a combination of pretreatment temperature and time. Glucose yields can be maximized at high DA pretreatment severity (i.e., high pretreatment temperature and/or long pretreatment time), but xylose degradation products always accompanied such conditions. Consequently, DA pretreatment condition in this study was selected at 130°C (20 psi) for 40 min, which falls into suboptimal DA pretreatment conditions to minimize xylose degradation and maintain high sugar yields [15], [22], [23]. No inhibition during saccharification was observed under the DA pretreatment condition in the present study.

By using COSLIF, the overall glucan release showed no correlation with lignin levels (R^2^ = 0.036) (**Fig. 2C**). The overall glucan release (L_1_+L_2_) from COSLIF-pretreated solids was ∼80–90% regardless of lignin levels. If only the solid part after pretreatment (stream L_2_) was accounted for, a similar strong correlation was observed in DA-pretreated solids, while enzymatic hydrolysis of COSLIF-pretreated solids had a weak negative correlation with lignin levels (**Fig. 2D**).

No correlation between lignin levels and xylan releases was observed from untreated biomass (**Fig. 2E**). The overall xylan releases (L_1_+L_2_) from COSLIF- and DA-pretreated solids were ∼80–85% regardless of lignin levels. Enzymatic xylan releases of COSLIF- and DA-pretreated solids were ∼10–15% and 5–8% (**Fig. 2G**), respectively, regardless of lignin levels. These results showed that most hemicelluloses were fractionated by COSLIF and DA, which was due to the glycosidic linkages of hemicelluloses are more labile [24] and easily hydrolyzed by COSLIF and DA. However, no correlation between lignin levels and xylan releases (overall and enzymatic) was observed.

Total sugar release from untreated biomass samples was variable, ranging from 3% to 17% with no correlation with lignin levels (**Fig. 2H**), implying that the chemical structures of carbohydrates of genetically modified switchgrass samples were also modified. The overall total sugar release from DA-pretreated samples had a strong negative correlation with lignin levels (R^2^ = 0.695). For COSLIF-pretreated samples, overall total sugar release was high (∼85–92%), regardless of lignin levels (**Fig. 2I**). Enzymatic total sugar release from DA-pretreated biomass samples was negatively correlated with lignin levels (R^2^ = 0.605), while a weak correlation was observed for COSLIF-pretreated biomass samples (**Fig. 2J**).

DA and COSLIF pretreatments have different modes of actions. DA aims at removing hemicelluloses and lignin partially. This is in agreement with our results that ∼85–90% hemicelluloses were removed during DA. Dilute acid has been shown to dissolve lignin partially at elevated temperatures; however, dissolved lignin can redeposit on the surface of DA-pretreated samples [25], so to decrease substrate accessibility to cellulase [14]. Consequently, high lignin levels negatively impact both overall and enzymatic glucan and total sugar releases. Concentrated H_3_PO_4_ as a cellulose solvent in COSLIF disrupted highly ordered hydrogen bonding networks among cellulose chains, as previously shown by CP/MAS ^13^C NMR and FTIR [26]. Varying pretreatment temperature using concentrated H_3_PO_4_ greatly influenced the extent of hydrolysis of polysaccharides. Under a modest reaction temperature (e.g., 50°C), acid hydrolysis of cellulose by concentrated H_3_PO_4_ was weak. Consequently, most cellulose after COSLIF was maintained as a reactive solid for the following enzymatic hydrolysis. Elevated biomass dissolution temperature resulted in excessive hydrolysis of cellulose and hemicellulose to soluble sugars, resulting in a separation challenge between soluble sugars from the cellulose solvent and costly recycling of the cellulose solvent, as is the case with other concentrated acid methods such as H_2_SO_4_ and HCl. Therefore, relatively low COLSIF temperatures were preferred (i.e., 50°C) and data shown for this temperature. Also, such low biomass dissolution temperatures avoided hemicellulose and cellulose degradation (data not shown). The COSLIF-pretreated solids regardless of lignin content were very reactive, allowing low enzyme usage and reducing energy costs associated with lower reaction temperatures, while maintaining high overall glucose yields (>85%) in a short time (<24 h). In contrast, DA-pretreated solids had lower overall glucose yields even after 72 h enzymatic hydrolysis.

Here, using uniform switchgrass lines which might only differ in their lignin content and composition allows confirmation that COSLIF was efficient enough to efficiently isolate a wide variety of lignin contents in the pretreatment step, such that the negative effect of lignin on saccharification was minimized. On the other hand, DA mildly disrupted the lignocellulosic matrix and the effect of residual lignin still played a significant role in downstream saccharification.

In conclusion, it was found that the effect of lignin contents on biomass saccharification efficiency was largely dependent on the pretreatment methods used. Using structurally-uniform switchgrass plants which only differ in their lignin content and composition, we showed that COSLIF-pretreatment was more efficient than DA-pretreatment and that the COSLIF pretreatment was also less dependent upon the lignin-content of feedstock biomass. This study re-emphasizes the importance of lignin content, however more importantly, shows that efficient pretreatment can minimize the negative effect of lignin on enzymatic saccharification.
